# The Final Link: Tapping the Power of Chemical Genetics to Connect the Molecular and Biologic Functions of Mitotic Protein Kinases

**DOI:** 10.3390/molecules171012172

**Published:** 2012-10-17

**Authors:** Robert F. Lera, Mark E. Burkard

**Affiliations:** 1 Graduate Program in Cellular and Molecular Biology, University of Wisconsin, Madison, WI 53705, USA; Email: lera@wisc.edu; 2 Department of Medicine and UW Carbone Cancer Center, University of Wisconsin, Madison, WI 53705, USA

**Keywords:** chemical biology, protein kinases, separation of function, mitosis, cell division

## Abstract

During mitosis, protein kinases coordinate cellular reorganization and chromosome segregation to ensure accurate distribution of genetic information into daughter cells. Multiple protein kinases contribute to mitotic regulation, modulating molecular signaling more rapidly than possible with gene expression. However, a comprehensive understanding of how kinases regulate mitotic progression remains elusive. The challenge arises from multiple functions and substrates, a large number of “bystander” phosphorylation events, and the brief window in which all mitotic events transpire. Analog-sensitive alleles of protein kinases are powerful chemical genetic tools for rapid and specific interrogation of kinase function. Moreover, combining these tools with advanced proteomics and substrate labeling has identified phosphorylation sites on numerous protein targets. Here, we review the chemical genetic tools available to study kinase function and identify substrates. We describe how chemical genetics can also be used to link kinase function with cognate phosphorylation events to provide mechanistic detail. This can be accomplished by dissecting subsets of kinase functions and chemical genetic complementation. We believe a complete “chemical genetic toolbox” will ultimately allow a comprehensive understanding of how protein kinases regulate mitosis.

## 1. Introduction

Mitosis is the critical phase during the cell cycle in which the cell undergoes dramatic remodeling to equally partition duplicated DNA into identical daughter cells. Errors in this process can result in daughter cells with an unequal distribution of chromosomes (aneuploidy) or a single daughter cell with twice the number of chromosomes (tetraploidy). Aneuploidy and tetraploidy occur naturally in some human tissues and a few whole-human aneuploid states are non-lethal. However, these conditions are usually associated with inviability during development [1] and disease states in adults [2,3].

The mitotic program is typically executed within an hour, at a time when DNA is condensed and largely inaccessible to transcription factors [4]. For this reason, it is perhaps natural that mitosis is not regulated by RNA transcription, but rather by more rapid posttranslational modifications such as phosphorylation. Protein kinases perform critical cellular signaling functions by catalyzing the transfer of the γ-phosphate from ATP onto the hydroxyl group of a serine, threonine, or tyrosine of a target protein. Removal of the phosphate is catalyzed by protein phosphatases. The addition and removal of phosphates on a target protein can drastically alter local charge, which regulate protein conformation, enzymatic activity, or protein-protein interactions. Thus, this single enzymatic activity allows for diverse molecular signaling events required for much of the dramatic intracellular remodeling during mitosis.

Understanding how protein kinases regulate mitosis is not trivial. Protein kinases comprise 1.7% of the proteins encoded in the human genome [5] and ~200 are thought to be active during mitosis [6,7]. Fortunately, several kinase families have emerged as key mitotic regulators: the cyclin-dependent kinases, Polo-like kinases, Aurora kinases, NIMA kinases, and the checkpoint kinases (Bub1, BubR1, Mps1) [8]. Moreover, important findings from genetically tractable organisms have provided a wealth of knowledge about human biology because of the high degree of functional evolutionary conservation in the mitotic program. Nevertheless, important differences between human cells and lower organisms have already emerged and these are proving to be important when contemplating kinases as therapeutic targets for human disease. Interrogating and providing a detailed understanding of kinase function during human mitosis has proved challenging.

Genetic manipulations, such as gene deletion or knockdown, offer powerful and specific strategies to elucidate functions of mitotic genes. However, the slow onset of action and irreversibility limit the utility of these techniques in probing the dynamic and distinct activities of protein kinases that occur during the brief mitotic window. Moreover, when contemplating functions of a protein kinase, it is important to distinguish its catalytic and structural roles. Although both roles are of interest, only its catalytic functions can be attributed to molecular phosphorylations. Conversely, the functional consequence of genetic deletion can be masked by homologs, which can supply essential kinase functions. For example, chemical inhibition, but not gene knockdown, of Cdk2 significantly inhibits cell cycle progression [9,10]. Thus chemical inhibition of a kinase (more relevant to pharmacologic therapy) can yield remarkably different outcomes than genetic removal. 

**Figure 1:**
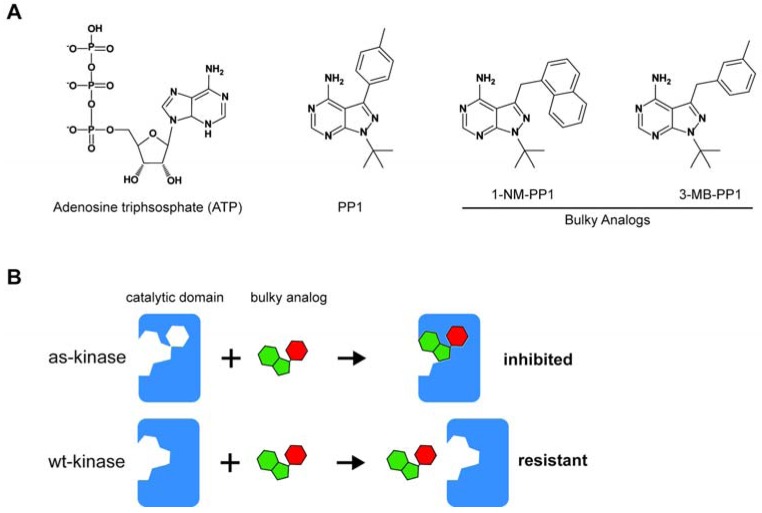
Chemical-genetic strategy for specific kinase inhibition. (**A**) Chemical structures of Adenosine triphosphate (ATP), the Src inhibitor PP1, and its bulky analogs, 1-NM-PP1 and 3-MB-PP1; (**B**) An enlarged ATP-binding pocket in the catalytic domain of the analog sensitive kinase (as-kinase) accommodates the analog, inhibiting activity, whereas the non-enlarged pocket of the wildtype kinase (wt-kinase) is resistant.

In contrast to genetics, chemical inhibitors of kinases allow rapid interrogation of catalytic function without altering kinase expression. These chemicals typically act by competitive inhibition of the ATP-binding domain to disrupt catalytic function. However, this strategy often has limited specificity because of high conservation of the ATP-binding domain across members of the human kinome. For this reason, off-target activity is encountered among pharmacologic kinase inhibitors, most often among kinases within the same family [11,12]. Nevertheless, inadvertent attribution of non-specific inhibitor effects to an incorrect target can be minimized. First, structurally unrelated compounds specific to one kinase, but with non-overlapping off-targets can be used in parallel [12]. Second, enzyme kinetic modeling may reveal conditions to allow for selective inhibition even when kinases have similar inhibitory profiles [13]. Finally, the kinase may be mutated to accept non-natural ligands to inhibit activity [14,15]. This chemical-genetic approach is accomplished by mutating the kinase “gatekeeper” residue to a smaller one such as glycine, to enlarge the ATP-binding pocket, which can then accommodate a bulky ATP analog as a competitive inhibitor. These analogs are typically pyrazolo-pyrimidine (PP) derivatives (Figure 1A) originally discovered as inhibitors of Sarcoma kinase (Src). By adding bulky napthylmethyl or methylbenzyl sidechains, these compounds lose their ability to disrupt Src activity. Because most wildtype protein kinases lack a glycine in the gatekeeper position of the binding pocket, they are insensitive to these ATP analogs (Figure 1B). Thus, the catalytic functions of the ‘analog-sensitive’ kinase may be interrogated in a rapid, reversible manner, with a specificity that is ensured by encoding the mutation genetically. 

Remarkably, this chemical-genetic approach has been used successfully for many protein kinases. Since its introduction, analog-sensitive alleles have been generated to interrogate the functions of mitotic kinases in diverse model systems: Cdk1/Cdc2 [16,17,18], Cdk7/Crk1 [18,19], Aurora B/Ark1 [20,21], Mps1 [22,23,24] and Plk1/Cdc5/Plo1 [18,25,26]. As outlined below, this chemical genetic approach also provides powerful tools to identify kinase substrates. But establishing links between kinase function and specific substrates remains a challenge. In this article, we review the strategies used to identify the substrates of mitotic kinases and comment on their strengths and limitations. We also address the barriers to linking substrate phosphorylation with biologic function and propose employing advanced chemical genetic techniques to help overcome them.

## 2. Strategies to Identify the Substrates of Analog-Sensitive Mitotic Kinases

Technological advances in phosphopeptide enrichment, substrate labeling and mass spectrometry have allowed for unbiased, high-throughput identification of protein targets for a handful of mitotic analog-sensitive (as) kinases (Table 1). For a comprehensive analysis of both targeted and unbiased strategies used to identify substrates of mitotic and non-mitotic as-kinases, we direct readers to the excellent review by Koch and Hauf [27]. Here, we limit our discussion to two predominant strategies employing as-kinases for unbiased discovery of the protein kinase substrates. We address caveats to interpretations of the findings, including the unique constraints imposed by rapid changes in intracellular state during mitotic progression. 

**Table 1:** Unbiased screens for protein targets of mitotic kinases using as-alleles.

Strategy	Authors	as-kinase	Organism	Proteins Identified
Chemical inhibition in intact cells	Oppermann *et al.* [28]	Plk1	human	382
	Koch *et al.* [21]	Ark1/Aurora	fission yeast	42
	Holt *et al.* [29]	Cdk1	budding yeast	308
Substrate labeling in cell extracts	Hengeveld *et al.* [20]	Aurora B	human	58
	Blethrow *et al.* [30]	Cdk1	human	>70
	Larochelle *et al.* [31]	Cdk7	human	7

### 2.1. Substrate Identification using Chemical Inhibitors

One powerful method to discover protein targets of individual mitotic kinases uses Stable Isotope Labeling by Amino acids in Cell culture (SILAC, [32]). With this method, intact cells are metabolically labeled with either amino acids containing heavy isotopes (e.g., ^15^N, ^13^C) or more common light isotopes (^14^N, ^12^C). The heavy-isotope labeled cells are then exposed to the kinase inhibitor whereas the light-labeled cells are treated with inert solvent. Afterwards, the two sets of cells are pooled, lysed, and trypsinized into peptide fragments. Phosphopeptides are enriched by affinity chromatography, analyzed by tandem mass spectrometry, and assigned to proteins. By pooling the two cell sets, a single mass spectrometry analysis can allow direct comparison of phosphopeptide quantities; a comparison that is far more precise than could be achieved with separate runs. Yet the different masses of the heavy and light amino acids readily allow the phosphopeptides from the two samples to be distinguished. The sequence of the peptide identifies both the protein and the site of phosphorylation on the protein. Kinase targets are identified by significant reductions in phosphopeptide quantities in the inhibitor group. To date, only a few studies have used this approach with analog-sensitive kinases (Table 1, top). 

Oppermann *et al.* [28] recently studied Plk1^as^ in human epithelial cells. Cells were arrested in mitosis using nocodazole and challenged with the ATP analog, 3-MB-PP1. In the control group, 3-MB-PP1 was washed out for the final 30 minutes prior to collection. Limiting hits to phosphopeptides containing the Plk1 consensus motif and exhibiting a two-fold increase in the washout group in at least two of four replicates, they identified 148 unique phosphorylation sites. In a separate analysis with less stringent requirements for consensus motif and replicate appearance, 382 proteins were identified as Plk1 downstream targets. A genetically matched line expressing wildtype Plk1 (Plk1^wt^) verified that these proteins were not found due to off-target effects of the inhibitor.

Koch *et al.* [21] generated an analog-sensitive Aurora kinase (Ark1^as^) cell line in the fission yeast *S. pombe.* Pre-mitotic cells were arrested and then released in the presence or absence of the ATP analog, 1-NM-PP1. A second experimental strategy included a microtubule depolymerizer, methyl-2-benzimidazole carbonate (MBC), to capture phosphorylations triggered by improper kinetochore-microtubule attachments. Limiting hits to phosphopeptides exhibiting a significant reduction in the inhibitor group in at least one of two replicates, 70 phosphorylation sites were identified on 42 proteins. Of these, at least 21 proteins were likely direct substrates as they harbored phosphopeptides with the Aurora kinase motif (R/K-X-pS/pT).

Holt *et al.* [29] generated a Cdk1^as^ line in the budding yeast *S. cerevisiae*. Cells were cultured in the presence or absence of 1-NM-PP1 and collected at separate points during mitosis. Limiting hits to phosphopeptides containing the minimal Cdk1 consensus motif (pS/pT-P) and a 50% reduction in the inhibitor group in at least one of three replicates, they identified 547 phosphorylation sites on 308 proteins. Importantly, there was significant overlap between substrates identified in their study with Cdk1 substrates identified previously *in vitro*, validating the effectiveness of this analysis. 

#### Considerations

These studies highlight several important issues when screening for mitotic kinase substrates. First, bulky ATP analogs, like pharmacologic inhibitors, possess off-target effects [12]. Therefore, genetically matched lines expressing the wild-type kinase should be used to control for non-specific effects [21,28]. Second, subcellular localization is important for kinase specificity [33]. In the above studies, the kinase is inhibited in an intact cellular environment preserving spatial relationships between kinase and its protein targets. However, phosphorylations may occur only at specific mitotic timepoints or under specific circumstances (e.g., the absence of microtubule attachment to kinetochore [21]); results may be specific to the timepoint selected (*i.e.*, arrested in prometaphase). Third, many phosphopeptides identified in this approach are indirectly controlled by the kinase studied; this occurs when the analog-sensitive kinase regulates the function of downstream kinases. Using a less stringent kinase consensus motif may introduce indirect relationships involving a second kinase with a similar minimal motif [21]. In contrast, stringent adherence to the kinase’s consensus motif may preclude identification of bona fide substrates containing a variant motif [34,35]. Finally, many direct phosphorylation events may be biologically unimportant, especially as the cellular energy expenditure for phosphorylations is small [36]. In spite of these limitations, SILAC labeling and analysis of phosphopeptides is an outstanding tool to discover novel phosphorylation events that are dependent on the activity of a single kinase.

### 2.2. Substrate Identification Using Signature Labels

**Figure 2:**
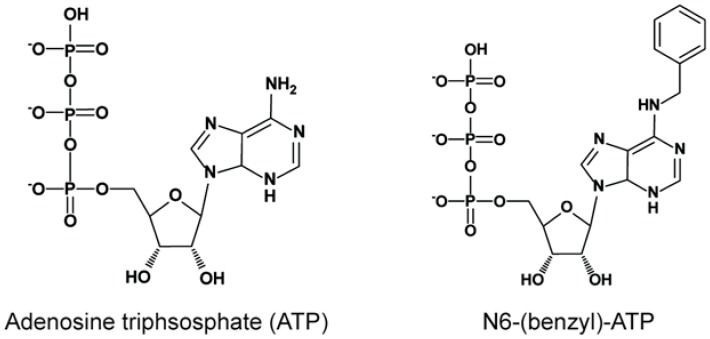
Chemical structures of ATP and the derivative N6-(benzyl)-ATP.

A second methodology to identify protein targets of analog-sensitive (as) kinases involves the direct labeling of substrates in cell extracts. In addition to accommodating ATP analogs to inhibit catalytic activity, as-kinases are capable of using bulky ATP derivatives (denoted A*TP), such as N6-(benzyl)-ATP (Figure 2) as phosphate donors, whereas wildtype kinases are unable to do so [37,38]. Exchange of the terminal phosphate of A*TP with a radioactive phosphate ([γ-^32^P]-A*TP) or thiophosphate (A*TP-γ-S) allows for signature labeling of substrates by the as-kinase. Because charged molecules do not readily cross the plasma membrane, these labeling strategies typically involve incubating whole cell lysates or extracts with recombinant as-kinase and A*TP. If [γ-^32^P]-A*TP is used, phosphorylated substrates are separated by gel electrophoresis, identified by autoradiography and excised prior to mass spectrometry identification. Fractionation steps prior to gel electrophoresis may be required to isolate lower-abundance substrates. Although this process has revealed new substrates for Cdk7 [31], the approach requires significant expertise and throughput is low. 

The use of A*TP-γ-S obviates the need for electrophoretic separation and excision. Instead, cell lysates are trypsinized to produce peptide fragments. Thiophosphorylated peptides are isolated by a covalent capture and release method [30], where thiol-containing peptides (those with cysteines or thiophosphates) are captured on iodoacetyl-agarose beads. Exposure to an oxidizing agent disrupts the phosphate diesters formed by thiophosphates, but not thioether linkages formed by cysteines, thereby releasing thiophosphorylated peptides from the beads. The resultant peptide mixture may then be analyzed by mass spectrometry for identification. This system has been used to identify over 70 Cdk1 substrates [30] and ~50 Aurora B substrates [20]. 

#### Considerations

The substrate labeling strategy has a distinct advantage over the inhibitor strategy–the relationship between kinase and substrate is direct since other kinases in the extracts cannot accommodate the bulky A*TP. However, some limitations should be noted. First, using A*TP-γ-S fails to identify phosphopeptides containing cysteine residues [30], whereas using [γ-^32^P]-A*TP requires multiple fractionation steps to isolate lower-abundance proteins. Second, cell lysis disrupts the spatial relationships between kinase and target proteins, allowing the kinase to artificially phosphorylate proteins that are ordinarily inaccessible. Finally, although analog-sensitive kinases have poor catalytic efficiency compared to wildtype counterparts [27], efficiency is markedly improved when using A*TP and can even exceed that of the wild-type kinase with ATP [31,37,39]. In such cases, direct substrate labeling experiments may introduce non-biologic kinase-substrate relationships. Despite these limitations, substrate labeling remains a remarkable tool for discovery of direct kinase substrates.

## 3. Current Strategies to Link Substrates to Kinase Function

Quantitative phosphoproteomic datasets, such as those generated in the above studies, provide an excellent foundation from which to explore kinase-substrate relationships; however, they provide little information regarding the biologic relevance of the relationships. Although functional relationships may be predicted using bioinformatics [40] these links must be verified experimentally. 

### 3.1. Classic Genetic Complementation: Replacement with Non-Phosphorylatable Mutants

An effective strategy to define a functional relationship between a kinase and substrate is to replace the endogenous wildtype substrate with a mutant non-phosphorylatable form. The first step involves mutating each identified phosphorylated serine/threonine on the substrate to a non-hydroxyl-containing residue (alanine or valine), preventing phosphorylation by the kinase. To confirm that no other residues are phosphorylated on the substrate, an *in vitro* kinase assay is performed with wildtype and mutant substrates. Finally a “knockdown/add-back” experiment is performed in which the candidate substrate is removed from cells (e.g., by RNAi) and the non-phosphorylatable mutant is added back. If a functional relationship exists between substrate and kinase, the non-phosphorylatable mutant should phenocopy kinase inhibition. Unfortunately, obtaining sufficient homogenous substrate knockdown and mutant expression in human cells can be challenging; therefore it is difficult to interpret the experiment if no phenotypic effects are observed. More importantly, it is an inefficient strategy to use to screen large sets of candidate substrates, requiring a candidate to be selected prior to initiating this set of experiments. Furthermore, it requires prior identification of phosphorylation sites. This can impair interpretation because of both false negatives and false positives. False negatives will occur when a candidate substrate is selected but one of a series of redundant phosphorylations has not been identified so that no phenotype is seen. Conversely, false positives occur when mutations inadvertently disrupt protein folding or function through removal of a critical hydroxyl group (the difference between serine and alanine side chains); the risk of this is significant when mutations are simultaneously introduced at multiple phosphorylation sites.

We conclude that genetic replacement is a powerful tool for understanding the functional significance of phosphorylation events but it is low throughput and results need to be interpreted cautiously. Moreover, kinases frequently perform multiple functions, so it may be difficult to reconcile effects of kinase function (observed by inhibition) with a large number of phosphorylations (observed with replacement strategies for individual substrates). Because of these considerations, we have sought intermediate tools to dissect specific kinase functions and to recover them with targeted re-introduction of activity.

### 3.2. Chemical Genetic Complementation: A New Twist

**Figure 3:**
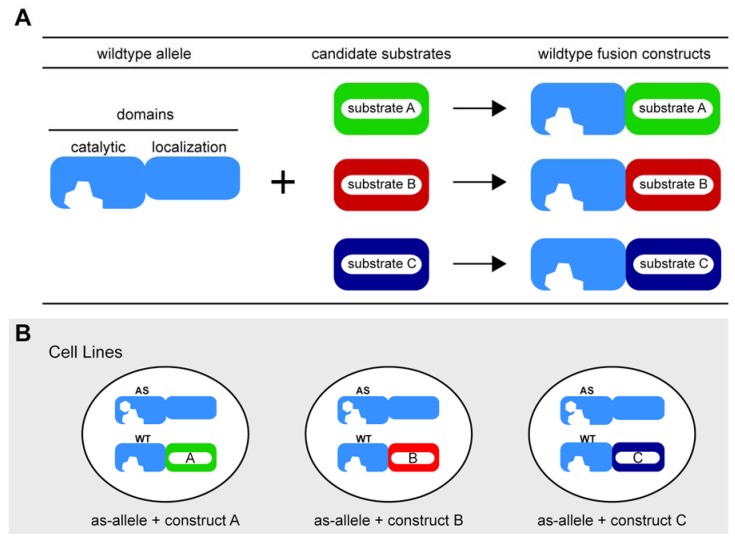
Chemical genetic complementation strategy to probe for functional kinase-substrate relationships. (**A**) Fusion constructs are generated by replacing the localization domain of the wildtype (WT) allele with candidate substrates; (**B**) Fusion constructs are introduced into cells expressing the analog-sensitive (AS) allele.

So the question remains how to quickly screen a large dataset of candidate substrates to identify functional relationships with the activating kinase. We propose a genetic complementation strategy that exploits the unique feature of the as-kinase system, namely the insensitivity of wildtype kinases to the bulky analog that inhibits the as-kinase (Figure 1B). For the kinase of interest, we propose creating a fusion construct containing the catalytic domain of the wildtype allele tethered to each candidate substrate (Figure 3A) and then individually introducing them into cells that express the as-allele (Figure 3B). The localization domain, if any, is expected to be unnecessary for these fusion constructs.

**Figure 4:**
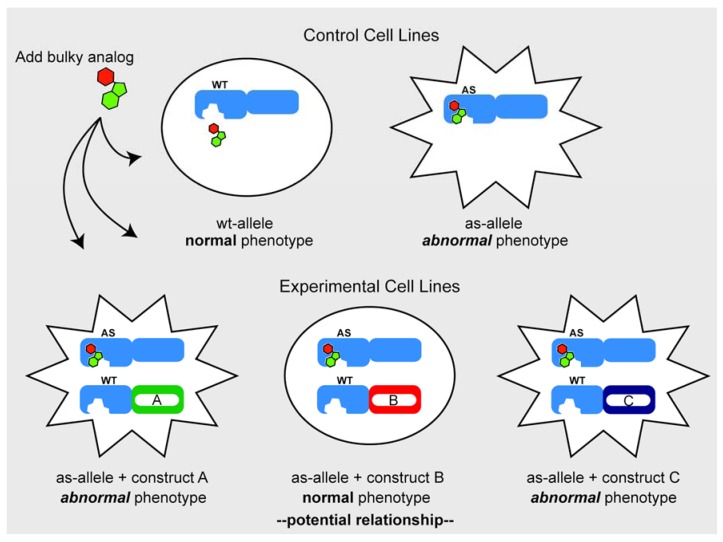
Assay for fusion construct complementation. Challenging cells with the ATP analog inhibits the as-allele, producing an abnormal phenotype (illustrated here as starburst cell shape). Note the wildtype (WT) allele is resistant to the analog, maintaining a normal phenotype. In cells expressing the fusion constructs, rescue of a normal phenotype suggests a functional relationship between the kinase and candidate substrate.

These fusion constructs are expected to localize specifically to areas where the endogenous substrate is present. The as-allele can be inhibited with the ATP analog, whereas the wildtype allele will remain active. Importantly, this activity will be restricted to the proximity of the substrate. In cells where an abnormal phenotype (*i.e.*, functional defect) is observed, the fusion construct is deemed to have failed complementation and the candidate substrate is considered unlikely to mediate that function (Figure 4, constructs A and C). Conversely, in cells where a normal phenotype is observed, the fusion construct is deemed to have complemented the as-allele, suggesting a relationship between kinase and substrate for that function (Figure 4, construct B). This is termed phenotypic rescue.

Although we are proposing phenotypic screens, fusing protein kinase domains to enforce localization has been used previously to illustrate the functions of several mitotic kinases. For example, such tethering has revealed the importance of reversible localization of the checkpoint kinase Mps1 and its yeast homolog, Mph1, to the kinetochore [41,42,43]. In another example, Aurora B kinase has been relocalized to distinct regions of the centromere and kinetochore to determine the location of the necessary activity to promote proper microtubule attachments to the kinetochore [44]. Finally, tethering Plk1 to proteins at the centrosome [45,46], kinetochore [47] and central spindle [48] delineates several of its multiple functions during mitosis. We propose using this protein tethering strategy to screen candidate substrates to narrow the candidate pool prior to embarking on detailed functional analysis of specific phosphorylations.

#### Considerations

To be a successful screening tool, the chemical genetic complementation approach we are proposing must minimize the errors inherent in screening tools: rejection of bona fide functional relationships (false negatives), and acceptance of functional relationships when indeed there are none (false positives).

As with the classic replacement strategy, we anticipate that false negatives are more likely to be encountered. In the chemical genetic approach, this may arise from several sources: the fusion construct may be poorly expressed, may localize incorrectly, may not be able to phosphorylate the substrate sufficiently, or it may introduce a non-physiologic dominant effect that obscures rescue. To overcome some of these challenges, a small affinity tag can be placed on the fusion construct. Fusion construct expression may be verified by immunoblotting with antibodies directed against the tag. Furthermore, antibodies directed against the candidate substrate will reveal expression of both substrate pools: the faster migrating endogenous substrate and the slower migrating substrate-kinase fusion construct. Fusion construct localization may be observed by immunofluorescence microscopy using antibodies targeting the affinity tag and the candidate substrate. Catalytic activity may be determined by immunoprecipitating the tagged fusion construct and then performing an *in vitro* kinase assay with a known substrate. Substrate phosphorylation by the fusion construct is more challenging to verify as developing and validating antibodies against specific phosphopeptide sequences is an expensive and time-consuming process. Nevertheless, substrate phosphorylation with fusion proteins has been demonstrated in several instances [45,48]. Alternately, it may be possible to detect substrate phosphorylation using a general phosphoserine antibody, with orthophosphate labeling, or in phosphorylation-dependent gel mobility shifts, which can be enhanced with special reagents such as Phos-Tag [49,50]. 

During screening, it is also possible to encounter false positives where we interpret a functional relationship when, in reality, no relationship exists. First, expression of the wildtype kinase may correct all defects regardless of the candidate substrate to which it is tethered. It is therefore important to screen multiple fusion constructs with unrelated candidate substrates to determine if this is the case. Second, overexpression of the candidate substrate may sufficiently correct the defect observed regardless of tethering to the wildtype kinase domain. This may be verified by overexpressing the candidate substrate without the kinase domain in inhibited cells. Alternately, a chemical inhibitor of the wildtype kinase may be introduced to block catalytic activity of the fused kinase. Finally, it is possible that the tethered kinase phosphorylates other nearby proteins so it is not assured that the candidate substrate is actually the one mediating biologic function. 

Thus, we propose tethering kinases to suspected substrates to narrow the candidate pool of substrates involved in a particular function. This is empowered by chemical genetics in which separate kinase alleles can be controlled independently [51]. Yet, employing this technique with multifunctional kinases may be particularly challenging.

## 4. Fractionating the Problem: Separating Multiple Kinase Functions

The approaches proposed here could be highly enlightening if a protein kinase had a single function, a single functional substrate, and many non-functional substrates. In nature, however, such unity in kinase function is rare. Most kinases have pleiotropic functions and multiple substrates making it challenging to isolate a single function for rescue by fusion-protein complementation. To overcome this, we propose using chemical genetic tools to dissect and isolate single biologic functions and then seek the cognate substrate for each. 

We have recently engaged in an effort to accomplish this with human Polo-like kinase 1 (Plk1) using multiple means of dissection including genetics, time, and thresholds of activity. For example, late mitotic functions of Plk1 can be delineated from early mitotic functions simply by inactivating this kinase at a specific time—this approach has revealed the important functions of Plk1 in triggering cytokinesis [25,48]. Moreover, we have discovered that discrete activities of Plk1 can be identified and separated through careful titration of kinase inhibitors, revealing that some functions require high activity, whereas others require low activity (Lera and Burkard, in editorial review). Genetic means of separating functions are also possible. For example, the Plk1^T210D^ mutant, previously thought to be constitutively active, exhibits a subtle recessive lethal phenotype in human cells, suggesting an inability to catalyze a specific essential phosphorylation [52]. Thus dissecting by time, activity, or genetic means can simplify the problem of pleiotropic kinases. Isolation of discrete kinase functions can empower the localization-by-tethering approach proposed above, by discovery of a single mediating phosphorylation.

## 5. Perspectives

It is difficult to isolate the specific signaling events of a single protein kinase to acquire a comprehensive understanding of how that kinase performs its essential cellular functions. Yet this information is necessary for the rational development and use of kinase inhibitors in the treatment of human disease. For mitotic kinases, the challenge is amplified because of multiple, often overlapping functions, an expansive substrate complement, and a brief window in which they exert their influence. 

Analog-sensitive kinases represent a clever chemical-genetic strategy to specifically interrogate the functions of single protein kinases. Importantly, this strategy provides both the genetic specificity and the temporal resolution necessary to probe specific events in mitosis. Moreover, it can be combined with advanced phosphoproteomics and signature-labeling strategies to generate large datasets of potential substrates. The final component is to integrate this information to elucidate the molecular details of how the mitotic kinase performs its essential cellular functions. This is where a major challenge lies.

As we have noted, large datasets of protein phosphorylations provide valuable, but incomplete information. Furthermore, the sheer volume of candidate substrates limits the utility (due to time and financial constraints) of traditional strategies such as phosphoantibody development or the “replacement-with-nonphosphorylatable-mutant” approach. We have proposed a two-pronged strategy for addressing this issue. First, fractionating the kinase’s functions by temporal, activity or genetic means narrows the functional window, thus limiting the number of candidate substrates involved. Second, performing chemical genetic complementation assays with kinase-substrate fusion constructs allows for efficient screening of the candidate substrate subsets. As with all screening strategies, there are limitations inherent in this approach. Nevertheless, chemical-genetic complementation expands the repertoire of strategies currently available. We hope that the complete “chemical genetic toolbox” will ultimately facilitate our understanding of how protein kinases perform their cellular functions and that this knowledge will empower rational development of kinase-targeted therapies. 
